# Progression and Metastasis of Lung Cancer: Clinical Features, Molecular Mechanisms, and Clinical Managements

**DOI:** 10.1002/mco2.70477

**Published:** 2025-11-14

**Authors:** Yunkui Zhang, Meixi Chen, Xumeng Fang, Yu Han, Yingke Li

**Affiliations:** ^1^ Department of Anesthesiology Shanghai Changzheng Hospital, Second Affiliated Hospital of Naval Medical University Shanghai China; ^2^ Department of Anesthesiology Shanghai Cancer Center Fudan University Shanghai China; ^3^ School of Life Sciences Shanghai University Shanghai China; ^4^ Shanghai Chest Hospital, Shanghai Jiao Tong University School of Medicine Shanghai China; ^5^ Department of General Thoracic Surgery China–Japan Friendship Hospital Beijing China

**Keywords:** bone metastasis, brain metastasis, liver metastasis, lung cancer

## Abstract

Lung cancer remains a leading cause of cancer‐related mortality worldwide, with metastasis leading to a poor prognosis. While advances in primary tumor management have improved survival, disease dissemination to distant organs, particularly the liver, bone, and brain, represents an unresolved therapeutic challenge. Metastasis is governed by complex interactions between tumor cells and the microenvironment, including immune evasion, angiogenesis, and organotropism. Current therapies often fail to address site‐specific molecular vulnerabilities or overcome physiological barriers such as the blood–brain barrier (BBB). A systematic review integrating clinical and mechanistic insights is urgently needed to guide translational efforts. This review synthesizes evidence on lung cancer metastases to three critical sites: liver metastases, where immunosuppressive niches and delayed diagnosis limit outcomes, and we emphasize the role of immune checkpoint inhibitors and liquid biopsies; bone metastases, characterized by osteolytic/osteoblastic lesions, which require biomarker‐driven therapies and multimodal pain management; and brain metastases, where BBB penetration and heterogeneity demand tailored approaches. By dissecting organ‐specific mechanisms, including circulating tumor cells, premetastatic niche formation, and metabolic reprogramming, this work highlights actionable targets for precision medicine. This review advocates for patient stratification and combination therapies to improve survival, offering a roadmap for future research on metastatic lung cancer.

## Graphical abstract

Lung cancer represents a significant global health threat. Despite progress in treatment, metastasis continues to present a major clinical challenge. This article reviews the metastases of lung cancer to the liver, bone, and brain, focusing on clinical symptoms, molecular mechanisms, and treatment strategies. Liver metastases from lung cancer underscore the need for early detection, rigorous monitoring, and prompt intervention. Enhancing systemic anti‐cancer immunity and reducing immunosuppression may improve therapeutic outcomes in these cases. Bone metastases highlight the critical role of biomarkers and the necessity of personalized and multimodal treatment approaches to enhance patient outcomes. For brain metastases, seen in both non‐small cell lung cancer and small cell lung cancer, a multidisciplinary treatment approach is essential. Future research must address immune evasion and strategies for overcoming the blood–brain barrier. Overall, this paper emphasizes the importance of understanding the pathways of metastasis to develop more effective treatments. It advocates for personalized treatment strategies targeting metastases at different sites.

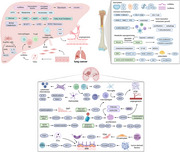

## 1 Introduction

Lung cancer is among the most prevalent malignancies worldwide and is a leading cause of cancer‐related death [1]. Its high morbidity and mortality rates represent a significant threat to public health. The disease primarily manifests in two histological forms: small cell lung cancer (SCLC), which constitutes 15% of cases, and non‐small cell lung cancer (NSCLC), which accounts for approximately 85% of cases [2, 3]. NSCLC can be further divided into three subtypes, large cell carcinoma, squamous cell carcinoma, and adenocarcinoma, with adenocarcinoma being the most common subtype [4, 5]. Recent advancements have greatly enhanced our understanding of lung cancer biology, the identification of predictive biomarkers, and the development of innovative treatment strategies. These treatment strategies include immune checkpoint inhibitors (ICIs) [6], targeted therapies [7], and improved early detection and screening techniques [8]. Medical progress has led to more personalized and effective treatments, resulting in better prognoses for many patients. However, challenges persist, particularly in the early detection of lung cancer. Most patients are diagnosed at advanced stages, which limits treatment options and often results in less favorable outcomes [9]. Therefore, implementing efficient screening methods, such as low‐dose computed tomography (CT) scans for high‐risk individuals, is crucial for increasing early diagnosis rates and reducing lung cancer mortality. The management of lung cancer has been transformed by the advent of molecularly targeted therapies, which have significantly improved outcomes for patients with specific genetic alterations, such as mutations in the anaplastic lymphoma kinase (ALK) gene [10] or the epidermal growth factor receptor (EGFR) gene [11]. Additionally, immunotherapy has demonstrated effectiveness in extending survival for patients with locally advanced NSCLC and those with advanced disease lacking targetable genetic mutations [12].

Lung cancer metastasis is the process by which cancer cells spread from the primary lung tumor to other organs and involves a complex sequence of events: local tissue invasion, entry into the bloodstream, survival in the circulation, exit from the bloodstream, and the establishment of a tumor in new organs [13]. This intricate process is regulated by various molecular programs that control cancer cell migration, survival, and proliferation [14]. The liver (24%), bone (39%), and brain (30%) are the most common metastatic sites of lung cancer, all of which are associated with shorter survival (medians: 5–6.8, 13, and <12 months, respectively). Liver metastasis occurs when lung cancer cells spread to the liver, typically via the bloodstream, where they form new tumor foci [15]. This process can lead to severe liver dysfunction and is frequently observed in advanced stages of lung cancer [16, 17]. Bone metastases involve the spread of cancer cells to the skeletal system, resulting in bone pain, fractures, and spinal cord compression [18]. These complications are often associated with a poor prognosis and require targeted treatment to manage symptoms and improve patients’ quality of life [19]. Brain metastases arise when lung cancer cells cross the blood–brain barrier (BBB) and infiltrate the brain tissue [20]. This condition can lead to neurological symptoms such as seizures, cognitive impairment, and increased intracranial pressure [21, 22]. The unique anatomical and physiological properties of the central nervous system present additional challenges in managing brain metastases, necessitating specialized treatment approaches (Table 1).

**TABLE 1:** Epidemiological characteristics of metastatic sites of lung cancer.

Metastatic site	Prevalence in all cancers (%)	Prevalence in lung cancer (%)	Median overall survival (months)	Data source
Liver	5.14	>5 per 100,000	4 (with) vs. 8 (without)	Horn et al. [23]
Bone	5.10	57.70	3 (with) vs. 4 (without)	Ryan et al. [24]
Brain	10–40 (among solid tumors)	SCLC (overall 15.83, metastatic 23.46); NSCLC adenocarcinoma (14.44, 26.82); squamous cell (5.29, 15.86)	NSCLC: 6 (adenocarcinoma), 4 (squamous cell), 10 (bronchioloalveolar); 6 (SCLC)	Lamba et al. [25]

This review systematically explores the molecular mechanisms and clinical manifestations of lung cancer metastasis to improve our understanding, with a focus on dissemination to critical organs such as the brain, liver, and bones. First, we examine the biological drivers of metastasis, including tumor cell‐intrinsic pathways and microenvironmental interactions. Next, we discuss the clinical challenges posed by organ‐specific metastases, such as BBB penetration in brain metastases and immunosuppressive niches in liver metastases. We then evaluate current therapeutic strategies, highlighting the limitations of conventional treatments and emerging approaches, including targeted therapies and immunomodulation. Finally, we explore how precision medicine, leveraging genomic profiling, liquid biopsies, and patient‐derived models, can enable tailored interventions. By integrating mechanistic insights with translational applications, this work aims to guide future research and improve the outcomes of patients with metastatic lung cancer.

## 2 Fundamental Biology of Lung Cancer Progression and Metastasis

The epithelial‐to‐mesenchymal transition (EMT) [26] increases tumor cell mobility and aggressiveness, whereas mutations in EGFR increase the liver metastasis risk [27], with vascular endothelial growth factor (VEGF) [28] and basic fibroblast growth factor [29]‐driven angiogenesis being crucial for metastatic tumor survival and growth in the liver [30]. Tumor‐derived exosomes promote liver metastasis by polarizing macrophages toward an immunosuppressive M2 phenotype [31] and suppressing NK cell [32] and T‐cell activity [33]. Exosomal microRNAs (miRNAs), such as miR‐1290 and miR‐126 [34], create a protumor hepatic microenvironment [35] through transfer.

EGFR [36], ALK [37], and Kirsten rat sarcoma (KRAS) [38] drive tumor progression by increasing cell survival and proliferation in bone metastasis. Noncoding RNAs (ncRNAs) have important roles in the development of bone metastases of lung cancer. miRNAs regulate lung cancer bone metastasis through distinct mechanisms, such as the targeting of SMARCA by miR‐660‐5p [39] that leads to the promotion of osteolytic metastasis. Long noncoding RNAs (lncRNAs) also play crucial roles in bone metastasis; for example, the lncRNA HOTAIR promotes osteoclast differentiation via exosomal transforming growth factor‐beta (TGF‐β)/parathyroid hormone‐related protein (PTHrP)/RANKL signaling [40].

SCLC can be molecularly classified into four subtypes, SCLC‐A, SCLC‐N, SCLC‐P, and SCLC‐Y [41], each of which have distinct immunotherapy responses and metastatic potential [42]. During local invasion, tumor cells breach the basement membrane [43]. Metastasis involves circulatory dissemination mediated by VEGF [44] and chemokines [45]. EGFR [46]/ALK [47] gene rearrangements significantly promote NSCLC distal metastasis and affect the treatment response. The heparin‐binding growth factor midkine promotes tumor invasion [48] and metastasis by activating the PI3K/Akt [49] and MAPK/ERK [50] pathways, driving tumor cell survival, proliferation, and DNA damage repair while inducing the EMT [51] via the Snail–N‐cadherin axis, leading to epithelial marker loss and mesenchymal marker gain.

## 3 General Mechanisms of the Metastatic Cascade

### 3.1 Role of Tumor Microenvironment Components

Interactions between tumor cells and various liver cell types facilitate the attachment, survival, and proliferation of cancer cells within the liver. Approximately 10% of liver cells are specialized macrophages known as Kupffer cells (KCs). They play dual roles in lung cancer liver metastasis and are essential for tumor cell clearance, immune suppression, and metastatic niche formation [52]. In early metastasis, KCs suppress tumor dissemination through NKG2D‐dependent tumor cell clearance [53]. In advanced stages, they promote metastasis by secreting interleukin‐10 (IL‐10) and expressing PD‐L1, creating an immunosuppressive niche [54]. Hepatic stellate cells (HSCs), which comprise approximately 15% of liver cells, are activated in response to angiogenesis [55] and immunosuppression [56]. HSCs are the main cells that deposit the extracellular matrix (ECM) during liver fibrosis [57]. Parenchymal hepatocytes, which constitute approximately 60% of liver cells and 80% of the liver mass, are crucial for metabolic functions, including serum protein production and detoxification [58]. The liver also contains various dendritic cell (DC) subtypes. A reduced DC frequency is significantly associated with liver metastases, an immunosuppressive microenvironment, and poor clinical outcomes [59]. Ultimately, the interactions between tumor cells and immune cells in the liver contribute to tumor evasion from immune surveillance and support the spread of cancer to other regions of the body [60].

The progression of bone metastasis is regulated by several immune mechanisms. The CXCL12/CXCR4 axis is crucial for fostering an immunosuppressive microenvironment that supports tumor growth and metastasis [61]. This axis helps cancer cells navigate to the bone marrow, where they can establish metastatic niches. Complement pathways, particularly the C5a/C5aR axis, are involved in immune responses [62]. When disrupted, this system can reduce the effectiveness of T‐cell responses, allowing metastatic cells to evade immune detection. An imbalance in signaling pathways such as the receptor activator of nuclear factor κB ligand/receptor activator of nuclear factor κB/osteoprotegerin (RANKL/RANK/OPG) system increases osteoclast activity, promoting bone resorption and metastatic growth [63]. Furthermore, the expression of the lncRNA MALAT1 affects the behavior of peripheral blood mononuclear cells, including their proliferation, autophagy, and T‐cell polarization [64].

The tumor microenvironment (TME) comprises diverse cellular and noncellular components that collectively promote tumor progression [65]. CD8^+^ T cells exhibit higher infiltration levels in metastatic brain lesions than in primary brain tumors, such as gliomas [66], but most of these cells are in a functionally exhausted state, with increased expression of inhibitory receptors such as PD‐1 and TIM‐3 [67]. CD4^+^ T cells are significantly altered in patients with oligometastases and are characterized by reduced numbers of early memory CD4^+^ T cells in peripheral blood and increased numbers of proinflammatory Th17 cells. Additionally, the levels of intratumoral infiltrating CD4^+^ T cells are correlated with metastasis patterns and are more abundant in oligosynchronous metastases than in polymetastatic disease [68]. Cancer‐associated fibroblasts secrete cytokines or chemokines to modulate immune cell activity [69]. Tumor‐associated macrophages and microglia (TAMs/Ms) drive immunosuppression by promoting invasion via matrix metalloproteinase (MMP)‐mediated ECM degradation [70] and angiogenesis through MMP/VEGF secretion [71]. Astrocytes support tumor growth through direct interactions that activate the Wnt/β‐catenin [72] and Notch pathways [73] and indirect immunomodulation, resulting in the release of TGF‐β/IL‐10 to suppress T‐cell activity [74]. Together, TAMs/Ms and astrocytes create an immunosuppressive TME that facilitates tumor survival and metastasis [75]. Additionally, lymphocyte activation gene 3 (LAG‐3) is highly expressed in depleted T cells, and blocking LAG‐3 can restore antitumor immunity [76]. The brain's cellular density, the pia mater, and the cerebrospinal fluid (CSF) help SCLC cells avoid immune system detection [77].

### 3.2 Metabolic Reprogramming for Metastatic Fitness

Metabolic reprogramming of lung cancer liver metastases involves several key molecular mechanisms. Dysregulated glycolysis and the Warburg effect, driven by ncRNAs [78], oncogenic pathways [79], and transcription factors [80], facilitate metastasis. Glycolytic inhibitors such as canagliflozin [81] or traditional Chinese medicine compounds [82] may have therapeutic value. A low level of GOT2 increases glutamine dependence, promoting metastasis by supporting nucleotide synthesis and glutathione production [83]. It protects metastatic cells from oxidative stress in the liver microenvironment. Chen et al. [84] showed that under glucose starvation conditions, lung cancer cells activate a PFKP–AMPK–ACC2 signaling axis to increase long‐chain fatty acid oxidation (FAO), thereby maintaining energy and redox homeostasis to promote survival. Mao et al. [85] linked BCAT1‐mediated branched‐chain amino acid catabolism to lung cancer metastasis via α‐KG production. This metabolite sustains SOX2‐dependent stemness, suggesting parallel roles in liver metastasis [85]. The synergistic effects of these metabolic pathways provide a key molecular basis for lung cancer liver metastasis.

Metabolic reprogramming of lung cancer bone metastases involves several key molecular mechanisms. Lung cancer cells undergoing bone metastasis exhibit a preference for aerobic glycolysis over oxidative phosphorylation (OXPHOS), where tumor cells preferentially convert glucose to lactate by upregulating glycolytic enzymes. This metabolic shift, which is exacerbated by hypoxia and acidosis in the bone microenvironment, promotes tumor proliferation and osteolytic progression [86]. Dysregulated cholesterol metabolism, as exemplified by the accumulation of 27‐hydroxycholesterol, promotes the generation of myeloid‐derived suppressor cells (MDSCs) [87]. Enhanced FAO stimulates PTHrP and IL‐6 secretion, activating osteoclasts through RANKL/NFATc1 signaling. This process drives osteolysis and the release of bone‐derived growth factors such as TGF‐β, accelerating tumor growth in bone [88]. Lung cancer bone metastases exhibit altered amino acid metabolism. Tryptophan‐derived kynurenine activates the aryl hydrocarbon receptor pathway [89], inducing osteoclast differentiation via c‐fos/NFATc1 upregulation and exacerbating bone destruction [90].

Metabolic reprogramming of lung cancer brain metastases involves several key molecular mechanisms. In lung cancer cells that have metastasized to the brain, AKR1B10 enhances the Warburg effect by upregulating LDHA, leading to lactate accumulation and inducing histone H4K12 lactylation. This process subsequently activates the transcription of the cell cycle‐related gene CCNB1, promoting DNA replication and cell cycle progression, ultimately resulting in resistance to pemetrexed [91]. Multiomics analysis revealed the significant activation of pathways related to mitochondrial metabolism in lung cancer brain metastasis [92]. The mitochondrial OXPHOS pathway is significantly upregulated in brain metastases, providing energy to support rapid proliferation. Targeting OXPHOS using drugs such as IACS‐010759 induces apoptosis in tumor cells [93]. The dual role of glutamine metabolism in lung cancer brain metastasis involves both promoting a malignant tumor phenotype through γ‐aminobutyric acid (GABA)‐mediated intracellular signaling and enhancing tumor adaptation by remodeling the TME. The inhibition of the FOXA2/ABAT axis by tumor cells reduces the catabolism of the downstream metabolite GABA, which in turn activates NF‐κB signaling. In addition, GABA promotes the activation of NLRP3 inflammatory vesicles in astrocytes, which further support brain colonization by the tumor cells [94]. Ngo et al. [95] revealed that brain metastases critically depend on PHGDH‐driven serine synthesis because of nutrient scarcity. PHGDH ablation selectively blocks brain colonization without affecting primary tumors, highlighting the presence of niche‐specific metabolic vulnerabilities [95]. In fatty acid metabolism, brain metastatic cells upregulate carnitine palmitoyltransferase 1a, promoting fatty acid β‐oxidation (FAO), which supplies energy to TAMs and sustains their M2 phenotype [96].

## 4 Summary of Molecular Mechanisms in Lung Cancer Metastasis

**FIGURE 1:**
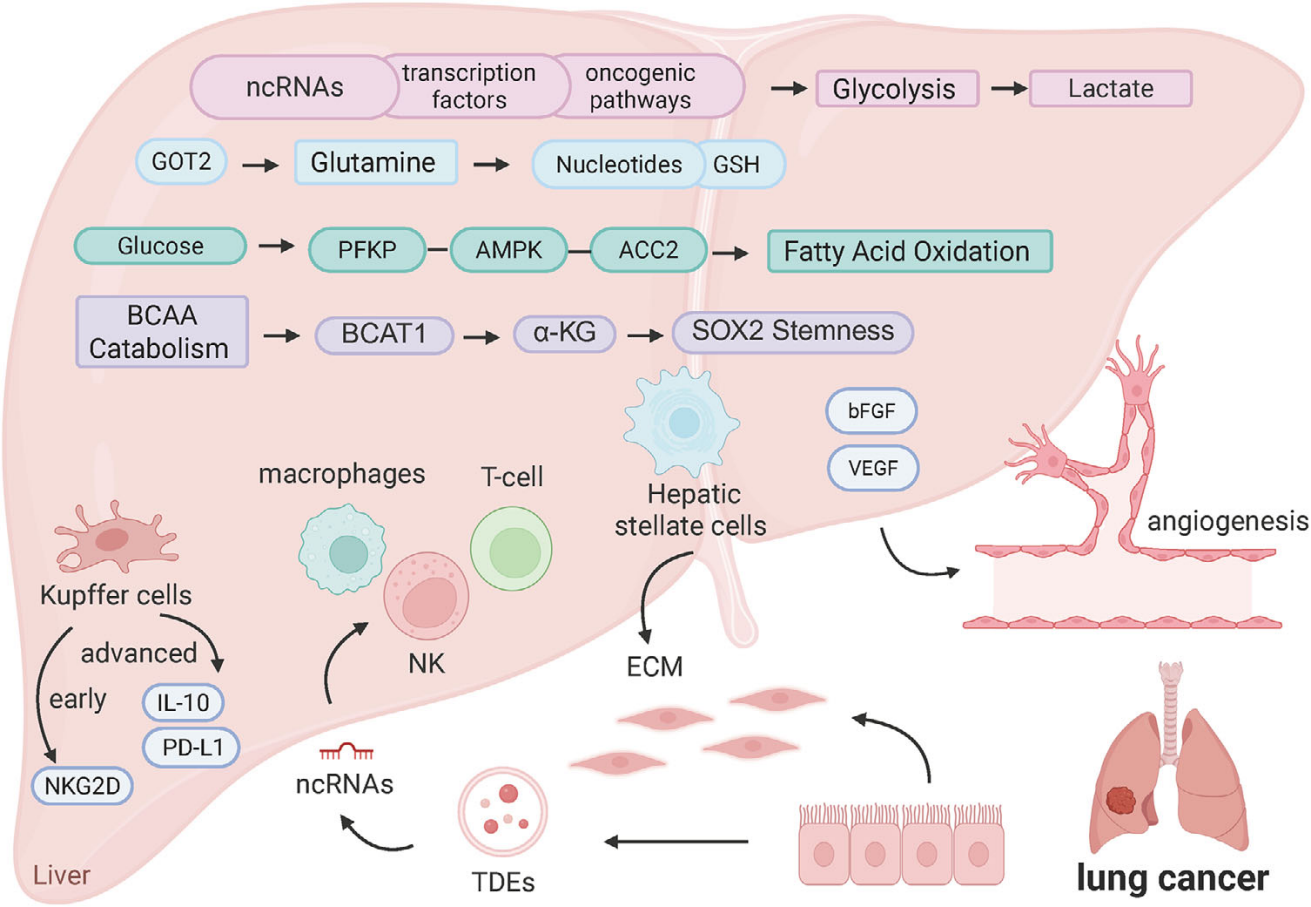
Molecular mechanisms of lung cancer liver metastasis. The development of lung cancer liver metastasis is stimulated by a well‐coordinated system that combines key metabolic processes, stromal cell activity, and immune cell interactions.

Liver metastasis of lung cancer is a major clinical concern because of its association with a poor prognosis. Secondary tumor deposits in the liver often lead to a range of debilitating symptoms that significantly diminish the patient's quality of life and frequently indicate advanced‐stage disease [97] (Figure 1). Although the precise triggers for liver metastasis are not fully understood, genetic mutations and changes in gene expression are believed to play significant roles. The molecular mechanisms driving liver metastases are intricate and involve a range of interactions between cancer cells and the liver environment. As an important metabolic organ in the human body, the unique metabolic microenvironment of the liver also provides metastatic cancer cells with unique survival advantages.

**FIGURE 2:**
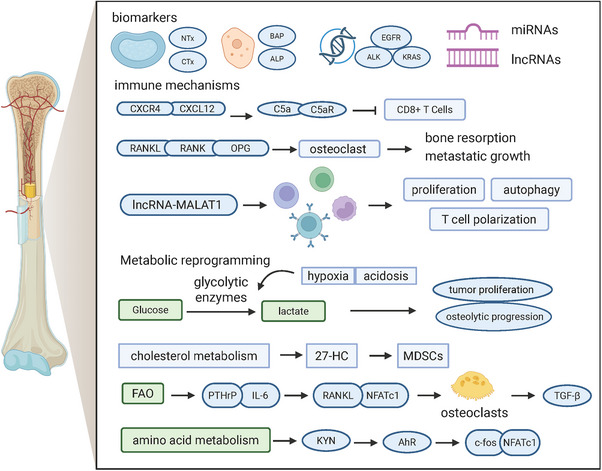
Molecular mechanisms of lung cancer bone metastasis. Tumor cells alter the microenvironment and activate osteoclasts through metabolic reprogramming and epigenetic regulation. These changes lead to lung cancer bone metastasis along with immunological regulation and hypoxia.

The emergence of bone metastases is a crucial phase in lung cancer progression and is frequently linked to a substantial deterioration of both the patient's quality of life and overall prognosis [98]. In more than 50% of cases, bone metastases indicate advanced lung cancer, particularly in NSCLC [99] (Figure 2). The molecular basis of the bone metastasis of lung cancer mainly involves complex interactions among biomarkers, immune responses, and metabolism. These pathways are critical for the initiation and progression of metastasis, as well as its spread to bone and further development.

**FIGURE 3:**
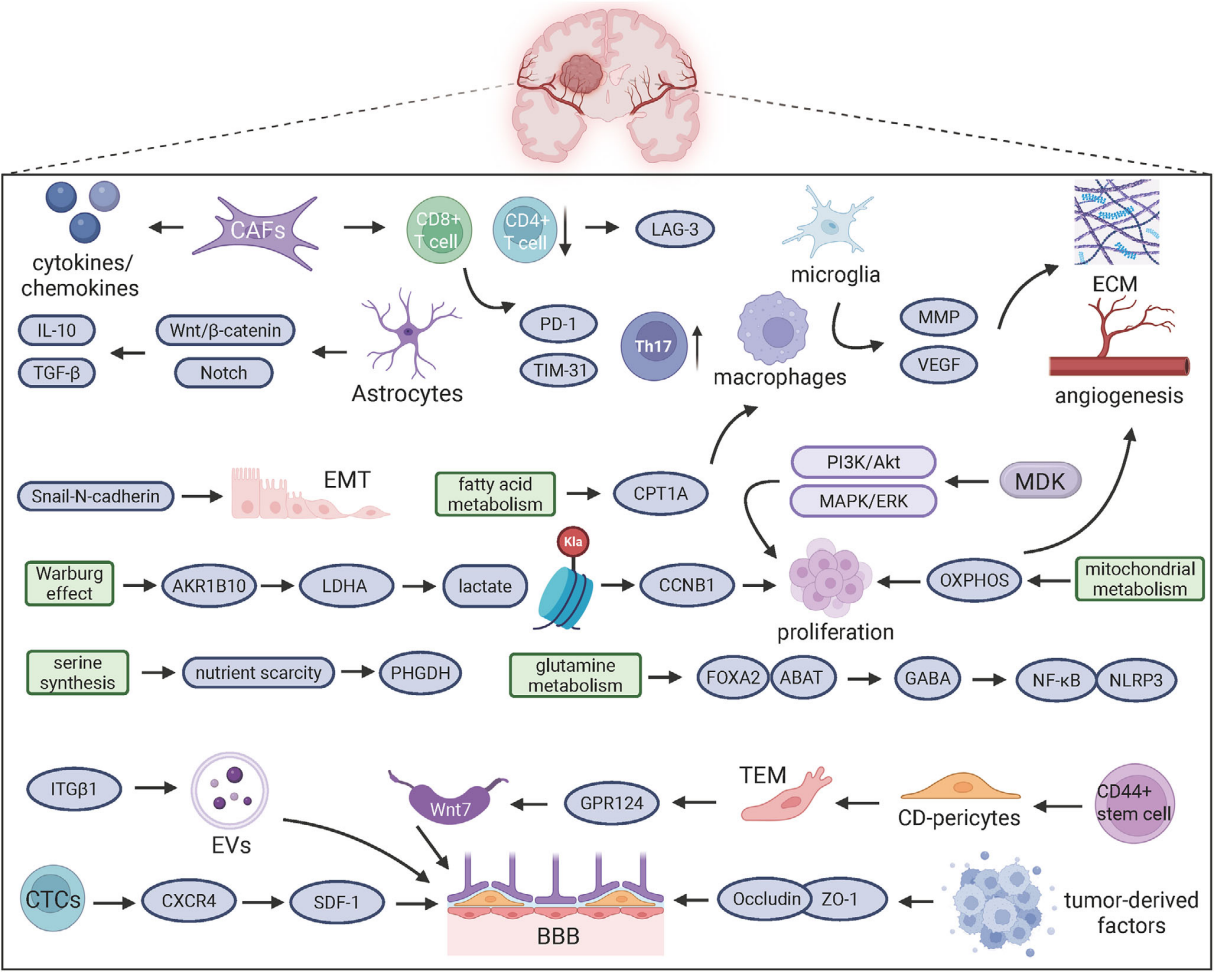
Molecular mechanisms of lung cancer brain metastasis The oncogenic pathways cooperate with metabolic reprogramming and immune checkpoint signaling to drive lung cancer brain metastasis. The model also includes the modulation of the blood–brain barrier (BBB) integrity.

Lung cancer‐related brain metastases present a critical clinical challenge and significantly affect patients’ prognosis [100]. Metastatic lung cancer often affects the central nervous system, particularly the brain, leading to severe symptoms that require urgent medical attention (Figures 3). The molecular mechanisms driving brain metastasis of SCLC and NSCLC involve complex, multistage processes characterized by distinct molecular alterations, immunity, metabolism, and the BBB. Tumor cells face a significant challenge in crossing the BBB, which is a key obstacle in their metastatic journey. CD44^+^ stem cells in lung adenocarcinoma (LADC) differentiate into pericyte‐like cells (CD‐pericytes), which exhibit significantly increased transendothelial migration. They penetrate the BBB and colonize the brain parenchyma by expressing GPR124, activating the Wnt7–β‐catenin signaling pathway [101]. Primary tumors release extracellular vesicles (EVs) that carry integrins, such as ITGβ1, that alter brain endothelial cell adhesion and increase BBB permeability, paving the way for metastatic cells [102]. Circulating tumor cells (CTCs) express CXCR4 and promote chemotaxis in response to high concentrations of SDF‐1 in the brain parenchyma for targeted brain metastasis [103]. The neovascularization of brain metastases is regulated by tumor‐derived factors, which downregulate the expression of tight junction proteins, such as ZO‐1 and Occludin, in endothelial cells, leading to increased BBB permeability. Aspirin can reduce BBB permeability by upregulating ZO‐1 and Occludin expression, but these effects can be reversed by TNF‐α [104].

## 5 Organ‐Specific Metastasis: Clinical Symptoms

### 5.1 Liver Metastasis

Lung cancer patients with liver metastases often experience neuralgia‐like symptoms, including abdominal pain or discomfort [105]. This discomfort arises when tumor cells spread to the liver, leading to secondary tumor sites and liver enlargement to accommodate the growing tumor load. Typically, patients report vague, persistent pain in the right upper abdomen, which can worsen with palpation or movement. In some cases, a palpable mass may be felt in the upper right abdomen, suggesting significant liver involvement. Additionally, liver metastasis can cause symptoms of liver dysfunction, such as jaundice, which manifests as yellowing of the skin and eyes due to extensive tumor invasion [106]. Elevated bilirubin levels can also lead to severe itching, as high bilirubin concentrations stimulate nerve endings in the skin [107].

Ascites, or the accumulation of fluid in the abdominal cavity, is another common consequence of liver metastases that can cause abdominal distension and pain [108]. In cases of lung cancer metastasis, extensive tumor invasion can impair liver function, leading to portal hypertension and an imbalance in fluid exchange between the intra‐ and extravascular spaces. Tumor cells may also invade the peritoneum directly, causing fluid leakage and an inflammatory response. Ascites can significantly impact a patient's well‐being, leading to symptoms such as dyspnea and early satiety, while also limiting their range of motion and overall quality of life. Additionally, tumors or other obstructions in the bile ducts can impede bile flow, leading to cholestasis [109]. This condition is characterized by dark urine and pale stools, resulting from elevated bilirubin levels in the blood due to bile duct blockage. The kidneys excrete excess bilirubin, darkening the urine, while the lack of bile pigment causes the stools to appear lighter. Prompt intervention is crucial to relieve the bile duct obstruction and prevent complications such as infection or liver failure [110].

Nonspecific symptoms such as fatigue, weight loss, and loss of appetite may indicate liver metastases in lung cancer patients [111]. These symptoms are often associated with the systemic effects of advanced disease. Tumors can create a high metabolic demand, which, combined with persistent anemia, malnutrition, or side effects from medications, can lead to fatigue [112]. Additionally, tumors can cause metabolic disruptions, decreased appetite, and problems with absorption and digestion, all of which can contribute to weight loss. Cancer patients may also experience weight loss because of the loss of fat and muscle tissue. Adverse effects of treatment, such as nausea, vomiting, or changes in taste, can further diminish appetite [113]. In some patients, the tumor may directly affect the appetite regulation center, exacerbating appetite loss, malnutrition, and weight loss.

The clinical presentation of liver metastases highlights the importance of early detection, careful monitoring, and prompt treatment. Given the aggressive nature of lung cancer and its potential for early spread, timely intervention is crucial. Addressing these symptoms effectively is essential for managing the disease, alleviating discomfort, and improving patient outcomes.

### 5.2 Bone Metastasis

Bone metastases of lung cancer present with a range of distressing symptoms. Severe bone pain is a prevalent issue and is typically described as deep, persistent, or intermittent pain that worsens with movement and often requires strong analgesics [114]. Pathological fractures frequently occur because of the infiltration of metastatic cells, which compromise bone structure [115]. These fractures, often resulting from minimal trauma, necessitate extensive treatment and rehabilitation and can be severely debilitating. Another major concern is spinal cord compression, which occurs when cancer cells invade the vertebrae, causing neurological symptoms such as tingling, back pain, paralysis, and motor weakness [116]. Similarly, nerve compression occurs when a growing tumor puts pressure on peripheral nerves, leading to movement disorders, altered sensory perception, and discomfort [117]. The bone‐lytic activity of metastatic cancer can also increase blood calcium levels, leading to hypercalcemia [118]. This condition can cause symptoms such as fatigue, confusion, constipation, and, in severe cases, coma [119]. Complications directly related to bone metastases, known as skeletal‐related events, often require palliative interventions such as radiotherapy or surgery to manage bone pain, stabilize bone structure, alleviate spinal cord compression, and address pathological fractures [120]. Patients with bone metastases typically face reduced mobility and functional decline, as structural damage and severe pain hinder daily activities [121]. Additionally, the psychosocial impact is significant, with many patients experiencing depression and anxiety, which further deteriorate their quality of life.

### 5.3 Brain Metastasis

SCLC accounts for approximately 14% of all lung cancer cases [122]. It is characterized by a high propensity for early metastasis, particularly to the brain, affecting approximately 10% of patients [123]. This early spread to the brain is a major contributor to mortality in affected individuals. Misdiagnosis as a primary brain tumor can be particularly dangerous because of the rapid growth of SCLC and its tendency to metastasize. Given that brain metastases of SCLC can lead to severe clinical symptoms, a multimodal treatment approach is often necessary. These treatments include surgery, radiation therapy, chemotherapy, and targeted therapies. Early detection and intervention are crucial for improving patient outcomes [124, 125]. One significant complication of NSCLC is leptomeningeal metastasis (LM), which can present in various clinical forms and often has subtle symptoms at the time of the initial diagnosis [126]. The incidence of LM in NSCLC patients ranges from 3.4 to 9.4%, with a higher frequency observed in subgroups with EGFR mutations and ALK rearrangements [127, 128].

The clinical presentation of brain metastases encompasses a variety of neurological symptoms, primarily resulting from elevated intracranial pressure and the direct invasion of brain tissue by metastatic lesions [129]. Frequent headaches, which are common symptoms, often indicate increased intracranial pressure [130, 131]. These headaches are typically severe and can worsen with physical exertion or changes in body position, significantly impacting the patient's quality of life. Additionally, patients may report back pain, which could be due to spinal cord compression or the involvement of nerve roots, presenting as localized or radiating pain [132]. Seizures may also occur, stemming from disruptions in the electrical activity of the brain caused by the tumor. Moderate to severe convulsions can severely affect both physical and mental health [133]. Additionally, metastatic cancer cells can invade specific brain regions, leading to localized deficits [134]. Such deficits may include impaired coordination, muscle weakness, or sensory changes, depending on the affected brain areas. Cerebral nerve paralysis resulting from brain metastases of lung cancer can present with symptoms such as vision loss, facial paresthesia, and muscle weakness [135, 136]. Visual abnormalities, including diplopia (double vision), visual field defects, or decreased vision, are commonly associated with damage to the optic nerve or visual pathways [137]. Diplopia results from dysfunction of the oculomotor nerve, causing overlapping images, whereas other visual impairments may stem from optic nerve compression or invasion. Hearing issues, such as diminished sensitivity or tinnitus, can indicate a compromised auditory nerve or pathways, potentially leading to significant hearing loss [138].

Cognitive impairment is a significant [139] and often debilitating symptom experienced by patients with brain metastases [140]. When metastatic lesions disrupt brain function, common issues include executive dysfunction [141], memory loss [142], confusion [143], personality changes [144], and disorientation [145]. Tumors affecting the speech region of the cortex can lead to speech and language difficulties, impacting patients’ ability to communicate [146]. Metastases in the occipital lobe or along visual pathways may cause visual disturbances such as double vision, cataracts, or even complete blindness. Motor dysfunction, including problems with balance, coordination, and movement, can further challenge mobility and independence. An irregular gait, characterized by shuffling or unsteady walking, increases the risk of falls and indicates nerve damage [147]. Additionally, patients may experience nausea and vomiting because of increased intracranial pressure [148]. The psychological impact of brain metastases is also considerable, with mood swings, anxiety, depression, and other behavioral disorders significantly affecting social relationships and mental health [149]. This complex array of symptoms underscores the need for a multidisciplinary treatment approach that addresses both the local treatment of brain metastases and the management of systemic symptoms. Additionally, a subset of patients may present with other symptoms, including limb discomfort, bladder or bowel dysfunction, aphasia, dysphagia, speech deficits, seizures, lethargy, neck stiffness, disorientation, and other speech disturbances [150].

## 6 Clinical Management of Metastatic Lung Cancer: Principles and Organ‐Specific Considerations

### 6.1 Integrating Local Therapies for Oligometastatic Disease

Treatments must be tailored to the needs of individual patients and the progression of their disease to effectively manage complications such as ascites and biliary obstruction resulting from lung cancer metastasizing to the liver. For ascites, treatment options may include medication to address fluid accumulation, peritoneal puncture to drain excess fluid and relieve pressure, and targeted therapy to treat the underlying lung cancer along with liver metastases [151]. In more severe cases, more invasive procedures, such as the placement of peritoneal dialysis tubes or surgical interventions, may be necessary to alleviate symptoms and improve the patient's quality of life [152]. Similarly, treatment for biliary obstruction should be customized based on the patient's specific condition. Options may include the placement of an endoscopic stent, percutaneous bile drainage, or direct surgical intervention.

When combined with systemic therapy or local radiotherapy, the use of chemotherapy as the primary first‐line treatment significantly prolongs patient survival [153]. The median overall survival (OS) of patients receiving first‐line chemotherapy is approximately 11.37 months compared with 8.5 months for patients treated with second‐line therapy and 6.00 months for patients receiving third‐line therapy [154]. Furthermore, in first‐line treatment, compared with chemotherapy alone, chemotherapy combined with antiangiogenic agents—particularly small‐molecule multitarget tyrosine kinase inhibitors (TKIs) such as anlotinib—has been shown to prolong progression‐free survival (PFS) [155].

Published research indicates that patients with liver metastases may exhibit a stronger response to ICIs [156]. This outcome has been observed in clinical trials using pembrolizumab and other inhibitors targeting programmed cell death protein 1 (PD‐1) and programmed cell death ligand 1 (PD‐L1) [157]. However, studies focusing on the immune system in the context of liver metastasis have revealed a tolerant environment that could reduce the effectiveness of ICIs. Initial clinical observations have shown that individuals with NSCLC and liver metastases respond significantly better to pembrolizumab [158]. Moreover, liver metastasis often leads to systemic immunosuppression through the production of CD11b^+^ inhibitory macrophages and regulatory T (Treg) cells. These cells contribute to distal immunosuppression by inducing the deletion of CD8^+^ T cells through mechanisms involving Fas ligand (FasL) [54]. Preclinical models, including genetically modified mouse models and isogenic cell line models, have been instrumental in elucidating this systemic immunosuppression [159]. These models suggest that treatments such as radiotherapy [160], combined checkpoint inhibition [161], and Treg cell clearance could increase the efficacy of immunotherapy [158] by addressing liver‐induced immunosuppression [162]. Recent translational studies in animal models have further explored these mechanisms. They reported that the activation of Treg cells suppresses distant immune responses and that CD11b^+^ inhibitory macrophages in liver metastases may clear CD8^+^ T cells through FasL [54]. These findings highlight the need for strategies to overcome liver‐associated immune suppression and improve therapeutic outcomes.

A multimodal approach is needed for the treatment of bone metastases of lung cancer because of the complex nature of the disease and its diverse effects. The current literature suggests a range of treatment strategies to address these challenges. These strategies are outlined in the following sections.

Advances in targeted therapies for lung cancer are largely influenced by the molecular mechanisms underlying bone metastasis. Key molecular variants in NSCLC, such as EGFR mutations [163] and KRAS mutations [164], as well as ALK rearrangements [165], are critical for the development of these therapies. Drugs such as afatinib [166], osimertinib [167], and other EGFR [168] TKIs are primarily used to target EGFR mutations. Compared with chemotherapy or first‐generation EGFR TKIs, osimertinib has shown superior efficacy in patients with T790M mutations, as shown in the AURA‐3 and FLAURA trials [169]. In addition to targeted therapies, ICIs such as anti‐PD‐1 antibodies have revolutionized cancer treatment by enhancing the body's immune response to tumors [170, 171]. The effectiveness of these inhibitors for treating bone metastases of lung cancer is currently under investigation. Research into how the bone microenvironment influences the immune response could lead to improved outcomes for patients undergoing immunotherapy for bone metastases [172].

Bisphosphonates [173], such as zoledronic acid [174], and RANKL inhibitors, such as denosumab [175], are typically used to manage bone metastases by reducing osteoclast activity and bone resorption. These treatments help to mitigate bone‐related complications and discomfort. However, as resistance to these existing treatments develops, research is increasingly focusing on new therapies specifically targeting bone metastases. Emerging options include cathepsin K inhibitors [176], sclerostin antibodies [177], and radiopharmaceuticals such as radium‐223 [178]. Given the diversity of lung cancer and the complexity of bone metastasis, researchers are exploring drug combinations to increase treatment effectiveness [179]. Combining TKIs with other targeted agents [180] or immunotherapies [181], along with the integration of bone‐modifying agents [182], may improve patient outcomes.

Despite brain metastases of SCLC diagnostic advancements, patients with SCLC and brain metastases generally have a poor prognosis, with a median OS of only 4.9 months [183, 184]. Managing brain metastases of SCLC is challenging because of the limited treatment options and the inherent resistance of this disease to standard therapies. Radiation therapies, such as stereotactic radiosurgery (SRS) [185, 186] and whole‐brain radiation therapy (WBRT) [187, 188], are typically used to control tumor growth and alleviate symptoms. However, these treatments are often associated with significant adverse effects, including neurotoxicity [189]. Chemotherapy is another option for treating brain metastases of SCLC, but its effectiveness is frequently compromised by the BBB, which hinders the delivery of therapeutic levels of the drugs within the brain [190]. This challenge highlights the urgent need for advanced therapeutic strategies that can effectively penetrate the BBB and target metastatic lesions [191]. Additionally, studies addressing the severe side effects of current chemotherapy drugs and developing more effective treatment options are needed [192]. The primary treatments for brain metastases of NSCLC include chemotherapy, stereotactic radiotherapy, whole‐brain radiotherapy, and surgical resection [193]. However, the effectiveness of traditional therapies is often limited by the BBB, which hinders the delivery of many chemotherapy drugs and affects the efficacy of radiotherapy and neurosurgery [77]. Consequently, these treatments may not significantly improve patients’ prognosis, particularly when intracranial lesions are involved. In light of these limitations, targeted therapy [194, 195] and immunotherapy [196] have emerged as promising new approaches for managing brain metastases in patients with NSCLC. Advances in understanding the molecular mechanisms of malignant tumors have facilitated the development of these innovative treatments, offering potential improvements in therapeutic outcomes for patients with brain metastases.

Recent advancements in immunotherapy have introduced new approaches for treating brain metastases of SCLC, significantly shifting the treatment paradigm [197, 198]. Current treatment options include ICIs and a combination of cisplatin and irinotecan. ICIs represent promising opportunities to improve patient outcomes by increasing the ability of the immune system to recognize and attack cancer cells [199, 200]. However, the responses to ICIs can vary among patients, highlighting the need for further research to identify specific prognostic indicators and refine treatment strategies. A thorough understanding of the underlying molecular mechanisms is essential for developing personalized treatments. This article emphasizes the role of biomarkers such as the potential benefits of immunotherapy [201, 202, 203] in combating brain metastases of SCLC. It also underscores the need for additional research to elucidate the function of specific molecules, such as S100A16 [204], in facilitating the spread of cancer to the brain and to discover new therapeutic targets that could transform treatment for SCLC patients. As our understanding of the TME has advanced, immunotherapy has emerged as a significant treatment option for brain metastases of NSCLC. ICIs have expanded therapeutic options for these patients [205]. Key immune checkpoints, such as PD‐1 and its ligand PD‐L1, play crucial roles in regulating T‐cell activity. The expression levels of PD‐1/PD‐L1 in the TME of brain metastases of NSCLC are strongly linked to the ability of tumors to evade immune detection [206]. Immunotherapy works by inhibiting the PD‐1/PD‐L1 signaling pathway, which can increase the activation of tumor‐specific T cells and exert antitumor effects [207]. Clinical research has demonstrated the effectiveness of PD‐1/PD‐L1 inhibitors, such as pembrolizumab and nivolumab, in treating patients with brain metastases of NSCLC [208]. These ICIs have shown the potential to have lasting effects on cerebral metastases and improve overall patient survival in some cases. Additionally, immunotherapy has been shown to increase the efficacy of other treatments, such as chemotherapy [209] and radiotherapy [210]. Radiotherapy can increase the immunogenicity of tumor cells and promote immune cell recognition and attack of tumors. Similarly, certain chemotherapeutic agents, such as cyclophosphamide, may increase the effectiveness of immunotherapy by reducing the number of immunosuppressive cells. This combined approach can potentially lead to improved treatment outcomes for patients with brain metastases of NSCLC.

The discovery of EGFR‐activating mutations [211] and ALK rearrangements [212] in NSCLC has significantly advanced the use of small‐molecule TKIs for treating advanced stages of the disease [213]. EGFR mutations are present in 10–20% of LADC patients [214], whereas ALK fusion genes are present in 3–7% of patients [215]. The development of TKIs targeting EGFR and ALK has notably improved the outcomes for patients with these specific mutations or rearrangements. In patients with NSCLC carrying EGFR mutations, first‐generation TKIs such as gefitinib [216] and erlotinib [217] substantially extend PFS and increase the objective response rate (ORR) by competitively inhibiting the ATP binding site of EGFR. Despite these benefits, these TKIs have limited effectiveness in treating brain metastases because of their poor BBB penetration. Second‐generation TKIs, such as afatinib [218], and ALK inhibitors, such as alectinib [219], have addressed this issue by irreversibly blocking their respective receptors, resulting in improved tumor control. Crizotinib [220, 221], a first‐generation ALK inhibitor, significantly improved the PFS of patients with ALK‐positive NSCLC. However, resistance to TKIs, particularly due to the T790M mutation of EGFR, has emerged as a significant challenge [222]. Third‐generation TKIs, such as osimertinib, have been developed to specifically target and inhibit the T790M mutation [223, 224]. Additionally, for patients who develop resistance to ALK inhibitors, second‐generation ALK inhibitors such as alectinib, ceritinib, and brigatinib provide new therapeutic options [225]. The challenge of treating brain metastases of lung cancer is compounded by the BBB, which often restricts the penetration of TKIs and results in inadequate drug concentrations in brain metastases. For instance, osimertinib [226] has shown an increased ability to cross the BBB and may represent a viable treatment option for patients with brain metastases of NSCLC. Additionally, several trials have demonstrated that combining TKI treatment with radiation therapy can exert synergistic effects. Radiation therapy may disrupt the BBB and increase TKI concentrations in the central nervous system, potentially increasing treatment efficacy [227]. However, further research is needed to identify the optimal combination of TKI and radiation therapy and to evaluate whether radiotherapy should be paused during TKI treatment [228].

### 6.2 Organ‐Specific Management Strategies and Challenges

The characterization of preclinical models, data from clinical trials and approved agents for liver metastases from lung cancer are shown above (Table 2). PDX excels in predicting the therapeutic response, GEMM excels in the mechanistic dissection of metastatic evolution, and CTCs facilitate liquid biopsy applications. All three trials share the commonality of immune‒chemotherapy combinations overcoming the limitations of traditional chemotherapy in prolonging survival. While ETER701 enhanced the clinical benefits through the addition of antiangiogenic therapy, this treatment resulted in increased toxicity. Conversely, the CASPIAN and IMpower133 regimens provide more manageable clinical profiles and are suitable for patients with poorer baseline conditions. While atezolizumab serves as a backbone immunotherapy, trastuzumab deruxtecan addresses oncogene‐driven resistance, and mRNA vaccines prevent micrometastatic progression. Key limitations include the biomarker dependency of atezolizumab, narrow targetability of trastuzumab deruxtecan, and individualized manufacturing constraints associated with mRNA vaccines.

**TABLE 2:** Therapeutic strategies for lung cancer liver metastasis: preclinical models, clinical trials, and approved agents.

Preclinical models		Clinical trials			Approved drugs	
Model type	Key features	Trial/regimen	Phase	Key findings	Agent	Class
Patient‐derived xenograft (PDX) [229]	Spontaneously metastasized in mice; retained the primary tumor TCA cycle labeling	ETER701 (benmelstobart + anlotinib + EC) [230]	III	Median OS: 19.3 vs. 11.9 months (HR 0.61); PFS: 6.9 vs. 4.2 months (HR 0.32)	Atezolizumab [231]	PD‐L1 inhibitor
Genetically engineered mouse model (GEMM) [232]	Drug‐resistant clone evolution; inducible multistage lineage tracing	CASPIAN (durvalumab ± tremelimumab + chemotherapy) [233]	III	mOS 8.6 vs. 13.9 months (LM−); HR 1.02 (95% CI 0.75–1.38)	Trastuzumab deruxtecan [234]	Antibody‒drug conjugate (ADC)
Circulating tumor cells (CTCs) [235]	Metastasis‐competent subsets; dynamic clonal persistence	IMpower133 (atezolizumab + chemotherapy) [233]	III	mOS 8.6 vs. 12.5 months (LM−); HR 0.73 (95% CI 0.51–1.11)	mRNA‐based personalized cancer vaccine [236]	Therapeutic vaccine

Existing treatment data indicate that the efficacy of cancer immunotherapy can be improved by increasing systemic anticancer immunity, reducing immunosuppression, and addressing liver metastases through radiation therapy or surgery. Moreover, interest in combining systemic immunotherapy with local treatments targeting liver metastases to improve clinical outcomes and overcome drug resistance is increasing. Clinical trials have explored strategies such as radiation therapy, combined checkpoint inhibition, and Treg cell clearance. These approaches show promise in improving responses to ICIs and mitigating the immunosuppressive effects associated with liver metastases.

The characterization of preclinical models, data from clinical trials and approved agents for bone metastases of lung cancer are listed above (Table 3). While 3D dormancy models facilitate studies of quiescence‐reactivating mechanisms, exosomal models decode EV‐mediated niche remodeling, and PDOs provide clinically actionable therapeutic predictions. Additionally, the three local interventions can delay resistance to systemic therapy but require an individualized balancing of efficacy and safety. Specifically, the CURB regimen is suitable for anatomically targeted intervention in oligoprogressive NSCLC, SINDAS provides a potentially curative strategy for EGFR‐positive oligometastatic disease, and the anlotinib combination improves the short‐term efficacy of antiangiogenic agents against SCLC. Critical distinctions include the requirement for c‐Met IHC testing with telisotuzumab treatment, the renal clearance limitations associated with zoledronic acid, and the efficacy of denosumab against lytic lesions. Combining ADCs for tumor control with RANKL inhibitors for bone stabilization represents an emerging therapeutic paradigm.

**TABLE 3:** Therapeutic strategies for lung cancer bone metastasis: preclinical models, clinical trials, and approved agents.

Preclinical models		Clinical trials			Approved drugs	
Model type	Key features	Trial/regimen	Phase	Key findings	Agent	Class
3D dormancy [237]	Murine bone metastasis model; retained primary tumor lipid metabolism	CURB oligoprogression (SBRT + systemic therapy) [238]	II	Median PFS: 10.0 months (SBRT) vs. 2.2 months (control); HR 0.41 (95% CI 0.22–0.75)	Telisotuzumab vedotin [239]	Antibody‒drug conjugate (ADC)
Exosomal regulation [240]	Tumor‐derived exosomal priming of bone niches; exosomal ncRNA‐driven osteolytic dominance	Anlotinib + etoposide/cisplatin or carboplatin [241]	II	Median PFS: 5.0 months (95% CI: 1.0–10.8); Median OS: 13.0 months (95% CI: 8.4–18.6)	Zoledronic acid [242]	Bisphosphonate
Patient‐derived organoids (PDOs) [243]	High‐fidelity modeling of bone metastasis; functional recapitulation of tumor biology	SINDAS (EGFR‐TKI + RT) [244]	III	Median PFS: 20.2 months (TKI + RT) vs. 12.5 months (TKI alone); HR 0.22 (95% CI 0.17–0.46)	Denosumab [245]	RANKL inhibitor

In addition to disease‐specific treatments, supportive care plays a crucial role in managing the symptoms and complications of bone metastases in patients with lung cancer. These approaches include pain management [246], treatment for bone complications [247], and other related services aimed at enhancing patients’ quality of life. Emerging therapies, such as those involving miRNAs [248] and lncRNAs [249], are being explored for their potential to regulate tumor growth and metastasis. These innovations may lead to the development of targeted drugs that directly influence the transcriptome, potentially transforming the treatment landscape for bone metastases.

Personalized therapy guided by tumor molecular profiling is becoming increasingly important for treating lung cancer with bone metastases. Effective management requires a comprehensive approach that combines bone‐modifying agents, immunotherapies, targeted treatments, supportive care, and the exploration of new strategies. Ongoing research aims to develop more effective and less harmful treatments, ultimately improving the outcomes of patients with this challenging condition.

The characterization of preclinical models, data from clinical trials and drug classes approved to treat brain metastases of lung cancer are shown in Table 4. Luciferase‐tagged cell lines offer high sensitivity for evaluating the dynamics of metastatic lesions and drug distribution but fail to recapitulate the BBB penetration process. Moreover, their intracranial inoculation artificially disrupts BBB integrity. In contrast, LRP1‐targeted PDCs demonstrate significantly enhanced BBB penetration rates while accommodating therapeutic payloads. However, their predominant reliance on single‐receptor mechanisms may overlook critical synergistic interactions among multiple barrier systems. Critical distinctions include the dependency of SRS on the lesion number, the requirement of the DLL3 biomarker for DeLLphi‐301, and the EGFR‐selective applicability of GAP BRAIN. These three agents share the commonality of precisely targeting driver gene alterations to prolong survival but exhibit distinct resistance mechanisms. Capmatinib resistance primarily arises from secondary MET kinase domain mutations, whereas lorlatinib resistance is associated with secondary ALK mutations. Osimertinib resistance typically involves MET amplification or histological transformation to SCLC.

**TABLE 4:** Therapeutic strategies for lung cancer brain metastasis: preclinical models, clinical trials, and approved agents.

Preclinical models		Clinical trials			Approved drugs	
Model type	Key features	Trial/regimen	Phase	Key findings	Agent	Class
Luciferase‐tagged cell lines [250]	Intracranial metastasis of luciferase reporter cells; subcutaneous NSCLC xenograft platforms	Stereotactic radiosurgery [251]	II	Neurologic death rate: 11.0 vs. 26.7% (historical WBRT controls); median OS: 10.3 months	Capmatinib [252]	MET tyrosine kinase inhibitor (MET‐TKI)
Blood‒brain barrier (BBB) [253]	LRP1‐targeted peptide–drug conjugates (PDCs); dual barrier‐penetrating shuttles	DeLLphi‐301 (tarlatamab monotherapy) [254]	II	Median PFS: 5.6 vs. 3.9 months; ORR: 45.3 vs. 32.6% (with vs. without brain metastases)	Lorlatinib [255]	ALK/ROS1 tyrosine kinase inhibitor (TKI)
Brain metastatic niche [256]	High perfusion and gray‒white matter junction preference; primary cancer‐specific tropism	GAP BRAIN (gefitinib + pemetrexed/platinum) [216]	III	Median intracranial PFS: 15.6 months (combination) vs. 9.1 months (gefitinib alone); HR 0.36 (95% CI 0.25–0.53)	Osimertinib [257]	Third‐generation EGFR tyrosine kinase inhibitor (EGFR‐TKI)

In conclusion, the complex clinical presentation of brain metastases of SCLC necessitates a multidisciplinary approach to diagnosis and treatment. While current therapies offer some benefits, the prognosis remains challenging, and less toxic and more effective treatment options are urgently needed. Continued research into the molecular mechanisms of brain metastases and the development of novel treatments have the potential to significantly improve outcomes for individuals with this severe disease. The treatment of brain metastases of NSCLC has evolved from relying solely on local therapies to embracing personalized, comprehensive approaches. Future therapeutic strategies will be focused on identifying molecular markers and integrating immunotherapy and targeted therapies to address specific mutations and resistance mechanisms. This tailored approach aims to increase treatment effectiveness and improve patient outcomes.

## 7 Diagnostic and Prognostic Biomarkers in Metastatic Lung Cancer

### 7.1 Imaging Biomarkers

Liver metastasis can cause biliary obstruction. The accurate diagnosis of biliary obstruction typically involves imaging techniques such as CT, MRI, or ultrasound. For a more detailed assessment, these imaging studies can be complemented by cholangiography, including endoscopic retrograde cholangiopancreatography. In addition, the diagnosis of bone metastases is often delayed because of the nonspecific nature of early symptoms, which can be mistaken for less severe conditions [258]. This misdiagnosis can lead to delays in crucial imaging and biopsy procedures. Advanced imaging techniques are typically needed to confirm the presence of bone metastases [259]. These techniques include bone marrow biopsy, X‐ray, CT, MRI, bone scans, and positron emission tomography. However, these procedures are costly and invasive and carry risks related to contrast agents and radiation exposure. The brain metastasis diagnosis requires both an evaluation of the clinical symptoms and diagnostic tests such as neuroimaging [260]. Brain metastases of SCLC are typically diagnosed using a combination of imaging techniques, including whole‐body bone scans [261], CT scans [262], and MRI [263]. These imaging modalities help determine the number, size, and location of metastatic lesions in the brain. Patients with brain metastases commonly present with a diverse array of neurological symptoms, reflecting the complex impact of metastatic spread on brain function. Identifying and implementing suitable treatment strategies for these symptoms are crucial to improve patients’ prognosis and quality of life.

### 7.2 Prognostic and Predictive Biomarkers for Specific Metastatic Sites

Blood tests may reveal abnormal liver function, with elevated levels of alkaline phosphatase (ALP), aspartate aminotransferase, gamma‐glutamyl transferase, and total bilirubin, providing diagnostic clues to the presence of liver metastases [264]. Given the serious clinical impact of bone metastases on lung cancer, identifying sensitive and specific biomarkers is crucial. Biomarkers found in tumor tissues, blood, or urine can help with early disease detection, monitoring the treatment response, and providing prognostic information [265]. Current research focuses on identifying biomarkers related to signaling pathways, bone formation, and resorption. Identifying these biomarkers could lead to more personalized and effective treatment approaches, significantly improving the early diagnosis and improving patient outcomes. The molecular mechanisms underlying bone metastasis in lung cancer involve a range of biomarkers. Bone resorption markers such as N‐terminal telopeptide [266] and C‐terminal telopeptide [267] reflect bone degradation, whereas bone formation markers such as bone‐specific ALP [268] and total serum ALP [269] indicate osteoblast activity. The brain metastasis diagnosis requires both an evaluation of the clinical symptoms and diagnostic tests such as CSF analysis [270]. A CSF analysis typically reveals alterations, including lymphocytosis, reduced glucose levels, elevated protein concentrations, and the presence of malignant cells [271, 272]. Although not all patients will exhibit all of these CSF changes, a perfectly normal CSF test is uncommon in patients with LM. This article emphasizes the role of biomarkers such as neuron‐specific enolase [273] in combating brain metastases of SCLC.

## 8 Current Challenges and Future Perspectives

### 8.1 The Promise of Multiomics and Artificial Intelligence for Personalized Management

In recent years, breakthroughs in single‐cell sequencing technology and the rapid development of multiomics integration strategies have provided a new perspective for understanding the molecular mechanisms of lung cancer metastasis [274]. scInfeR, a single‐cell multiomics annotation tool based on graph neural networks, integrates scRNA‐seq, scATAC‐seq, and spatial transcriptome data and has an F1 score of 0.94 in the annotation of 329 cell types, significantly improving the accuracy of recognizing rare cell subtypes [275]. The CancerSCEM 2.0 database includes 1466 new cancer single‐cell datasets covering 74 cancer types, provides metabolic flux inference and comparative cross‐tissue analysis capabilities, and supports studies of microenvironmental heterogeneity in metastatic lung cancer [276]. Izar et al. employed multiomics analysis to reveal the key molecular characteristics of NSCLC brain metastases. Their findings demonstrate that compared with primary tumors, metastatic brain lesions exhibit greater chromosomal instability (CIN) and contain a rare subpopulation of CIN‐high tumor cells with neural‐like transcriptional features. While present in small quantities in primary tumors, this subpopulation becomes significantly enriched in brain metastases. Additionally, this study revealed an immunosuppressive TME in brain metastases characterized by reduced CD8^+^ T‐cell infiltration, increased numbers of Treg cells, and reprogrammed myeloid cell populations [277].

### 8.2 Unmet Needs and Future Directions

The rapid development of organoid technology has allowed this model to achieve groundbreaking and innovative applications in the field of cancer metastasis research, especially in the accurate prediction of treatment responses. Wang et al. [278] constructed lung cancer organoids (LCOs) using malignant serous effusion with a 75.7% success rate and 70.5% genomic concordance with the primary tumor. LCO drug sensitivity assays predicted the clinical response to targeted therapies with 84% accuracy, such as the response to ositinib, providing personalized regimens for patients with advanced metastasis [278]. An individualized patient tumor organoid (IPTO) platform was developed by Peng et al. [279] as a personalized brain tumor biobank, where patient‐derived tumor explants were engrafted into iPSC‐derived cerebral organoids to model tumor–normal tissue interactions. In a prospective clinical study, IPTOs successfully predicted glioblastoma resistance to temozolomide, demonstrating superior accuracy to that of conventional MGMT promoter methylation analysis [279]. Liu et al. [280] combined patient‐derived primary LCOs with a modular microfluidic chip to achieve single‐organoid‐level immune response profiling. Their findings revealed that during αPD‐1 treatment, CD8^+^ T cells mediate tumor killing via PRF1/GZMB, while M2 macrophage infiltration suppresses T‐cell accumulation, contributing to immunotherapy resistance in brain metastases [280].

## 9 Conclusion

The importance of early detection and treatment in managing lung cancer cannot be overstated, as the complexity of treatment significantly increases once distant metastases develop [281]. Lung cancer metastasis to the brain, liver, and bones is particularly challenging because of its aggressive nature and profound impact on patients’ prognosis. The development of accurate metastasis models is crucial for effective detection and intervention prior to the onset of metastasis, although this process presents considerable challenges.

Lung cancer liver metastasis, bone metastasis, and brain metastasis are specific in terms of the molecular mechanisms, immunosuppression, and metabolic activities. Liver metastasis is characterized by metabolic adaptive advantages, and tumor cells achieve colonization and proliferation by hijacking the rich nutritional resources and unique immune‐exempt microenvironment of the liver [282]. As the metabolic center of the body, the liver has high concentrations of growth factors and immunosuppressive cell populations, providing a favorable environment for metastatic foci to survive [283]. The pathology of bone metastasis is characterized mainly by the formation of a “vicious cycle” [284]. Tumor cells activate osteoclasts by secreting factors such as PTHrP, which leads to the degradation of the bone matrix and the release of growth factors such as TGF‐β, thus forming a positive feedback loop that promotes tumor growth [285]. This bidirectional interaction gives bone metastases a unique self‐sustaining ability. The specificity of brain metastases, on the other hand, stems from the anatomical and physiological properties of the BBB, which constitutes an effective barrier to drug penetration through a tightly connected layer of endothelial cells and multiple efflux transporters [286]. Moreover, the unique immune‐exempt properties of the brain parenchyma further increase the therapeutic difficulty [287]. Tumor cells disrupt the integrity of the BBB by secreting protein hydrolases such as MMP‐9 but also limit the effective delivery of most therapeutic agents [288].

Future research will need to focus on developing more complex and sophisticated modeling systems to advance our understanding and treatment of lung cancer metastases. Despite the progress achieved with the current models, enhancing their practical relevance and accuracy will require interdisciplinary collaboration, the adoption of innovative modeling techniques, and ongoing refinement. These efforts aim to improve patients’ quality of life and prognosis by facilitating earlier and more effective treatments.

## Author Contributions

Yunkui Zhang and Meixi Chen were responsible for writing the manuscript and collecting the references. Xumeng Fang was mainly responsible for the revision of English grammar and expression. Yu Han and Yingke Li played a guiding role in and manuscript revision. All authors read and approved the final manuscript.

## Funding

This work was supported by grants from Natural Science Foundation of Shanghai (23ZR1477800 to Yingke Li) and the CAMS Innovation Fund for Medical Sciences (CIFMS, No. 2024‐I2M‐C&T‐B‐091).

## Ethics Statement

The authors have nothing to report.

## Conflicts of Interest

The authors declare no conflicts of interest.

## Data Availability

The authors have nothing to report.
